# Sleeping Beauty and the Story of the Bacille Calmette-Guérin Vaccine

**DOI:** 10.1128/mBio.01370-16

**Published:** 2016-08-30

**Authors:** Helen A. Fletcher

**Affiliations:** Department of Immunology and Infection, London School of Hygiene & Tropical Medicine, London, United Kingdom

## Abstract

*Mycobacterium bovis* BCG is the only available vaccine for protection against tuberculosis (TB). While BCG protects children from severe disease, it has little impact on pulmonary disease in adults. A recombinant BCG vaccine BCG Δ*ureC*::*hly* (strain VPM1002) is in advanced clinical trials and shows promise for improved vaccine safety but little change in efficacy in animal models. A second-generation recombinant BCG vaccine with an additional deletion of the *nuoG* gene (BCG Δ*ureC*::*hly* Δ*nuoG*) shows improved efficacy in a mouse model compared to that of VPM1002. BCG was first used in humans in 1921 and, like Sleeping Beauty pricked by the spinning wheel, we have slept for 100 years, showing a reluctance to invest in clinical development or in biomanufacturing capacity for TB vaccines. The advance of recombinant BCGs should awaken us from our sleep and call us to invest in new-generation TB vaccines and to protect the biomanufacture of our current BCG vaccine.

## BACKGROUND

Nearly 2 million people die each year of tuberculosis (TB), and an estimated one-third of the world’s population is infected with *Mycobacterium tuberculosis* (1). In 2015, TB was reported as the leading cause of death due to a single infectious disease (2). Improved diagnostics and shorter, less toxic drug treatments would help to reduce the global burden of disease, but a significant impact on the rate of TB disease can only be made with a more effective vaccine (http://www.who.int/tb/post2015_strategy/en/) (3).

Bacille Calmette-Guérin (BCG) is the only currently licensed vaccine for the prevention of TB. BCG is a live, attenuated strain of *Mycobacterium bovis* that was attenuated through passage of the organism in culture more than 230 times. Seed stock of BCG was distributed to other manufacturers, and the process of attenuation continued, resulting in more than 16 (4) distinct strains of BCG vaccine worldwide. BCG is one of the most widely used vaccines in the world, but its efficacy is highly variable. In a recent meta-analysis of the literature, it was shown that the efficacy of BCG is lower in those with previous exposure to environmental mycobacteria or to *M. tuberculosis* itself (as assessed by reactivity to the tuberculin skin test) (5). The efficacy of BCG in countries where TB is endemic is therefore highest in unexposed infants and lowest in adults, with an average efficacy of 50% in children and typically no efficacy in adults.

The importance of the Th1 pathway in protective immunity has been confirmed through multiple observations from human genetic studies and murine *M. tuberculosis* challenge experiments. In South African infants, the BCG-specific gamma interferon (IFN-γ) enzyme-linked immunosorbent spot assay (ELISPOT) response was associated with reduced TB disease risk over the following 1 to 3 years of life (6). This immune response was predominantly a CD4^+^ polyfunctional response, with little detection of antigen-specific CD8^+^ T cells at the time point tested (4 to 6 months of age) (6).

New TB vaccines seek to improve protection against TB either by increasing the magnitude of the CD4 T cell response induced by BCG or by broadening the immune response, for example, through the induction of a CD8 T cell response. Strategies for improved protection include whole-mycobacterial-cell-derived vaccines, virus-vectored subunit vaccines, and adjuvanted protein subunit vaccines (7). The subunit vaccines are typically given after BCG immunization to boost the immune response primed by BCG, whereas whole-mycobacterial vaccines can either be used as BCG booster vaccines or as a replacement for BCG. The current BCG vaccine is thus able to confer protection, but room for improved protection through boosting or broadening of immunity exists.

## RECOMBINANT BCG VACCINES

BCG Δ*ureC*::*hly* (strain VPM1002) is a recombinant BCG strain that has been modified by the insertion of listeriolysin and the deletion of urease (8). These modifications aim to enhance both the immunogenicity and the safety of the parental BCG vaccine strain. Listeriolysin is thought to perforate the phagolysosome, enabling leakage of mycobacterial antigen from VPM1002 into the cytosol and, thus, facilitating cross-presentation and the enhancement of a CD8 T cell response (8). VPM1002 enhances inflammasome activation and autophagy in C57BL/6 mice (9); mouse studies also showed an association of central memory CD4 T cells and T follicular helper cells with the increased protection (10). These immune responses are broader than those induced by the parental BCG strain, where a CD4^+^ Th1 response dominates. In early-phase clinical trials, the CD4^+^ and CD8^+^ antigen-specific responses to VPM1002 did not differ from those induced by BCG, although there was early enhancement of purified protein derivative (PPD)-specific antibodies (11).

Due to an enhanced safety profile in preclinical models, VPM1002 is being assessed as a BCG replacement vaccine for HIV-exposed infants. The risk of disseminated disease due to uncontrolled replication of the current BCG vaccine is higher in HIV-exposed infants, and therefore, BCG is not recommended in this population. Phase II trials are ongoing in South Africa and are due to start in India soon, with market entry expected within 5 years (http://www.tbvi.eu/wp-content/uploads/2016/02/Grode_SII-VPM-Status-on-TB-vaccine-150318.pdf). Phase III trials for prevention of the recurrence of TB disease in those previously treated for TB are also expected to commence in India within the next few years (http://indianexpress.com/article/explained/a-new-tb-vaccine-is-in-the-works-and-heres-why-india-is-excited/). If these clinical trials prove VPM1002 to be safe, even if not more effective against clinical disease, VPM1002 will be the first new TB vaccine to enter the market since 1921, when BCG was first used in humans.

Research with recombinant BCG strains continues (reviewed in reference 12). Gengenbacher et al. report that the inflammasome activation and autophagy induced by VPM1002 can be further enhanced through the deletion of *nuoG* (BCG Δ*ureC*::*hly* Δ*nuoG*) (13). The *nuoG* gene encodes NADH-quinone oxidoreductase and is involved in mycobacterial respiration. Deletion of this gene leads to enhanced apoptosis of the host cell during mycobacterial infection (14, 15). Gengenbacher et al. show that the protective efficacy of VPM1002 in the mouse model is further improved by the deletion of *nuoG* (13).

## IMPACT OF A NEW RECOMBINANT BCG VACCINE ON THE DEVELOPMENT OF VPM1002

If BCG Δ*ureC*::*hly* Δ*nuoG* is more effective than VPM1002, what are the implications for the current clinical development of VPM1002? BCG was first used as a human vaccine in 1921 and, like Sleeping Beauty pricked by the spinning wheel, we have slumbered for 100 years, depending on BCG alone for protection. The high risk of failure of TB vaccine candidates in costly, late-phase clinical trials and the low commercial value of a TB vaccine to industry are blamed in part for the sluggish pace of TB vaccine development (16). VPM1002 may be the first new-generation TB vaccine to enter the market as a safer alternative to the BCG vaccine in HIV-exposed infants. After the introduction of VPM1002, we should not sink back into our sleep for another 100 years but should capitalize on our expertise, iteratively improving VPM1002 and our other vaccine candidates, in addition to generating novel vaccine platforms. Through the clinical development of VPM1002 and other vaccine candidates, such as MVA85A, we have advanced our understanding of immune correlates and built capacity for clinical testing and biomanufacturing that will greatly accelerate the development process for 2nd-, 3rd-, and 4th-generation TB vaccine candidates.

BCG Δ*ureC*::*hly* Δ*nuoG* is our wake-up call, which has shown us that there is potential for further improvement on the protection induced by BCG and VPM1002. We should respond to this call for action with increased energy and investment in TB vaccine development.

## IMPACT OF A NEW RECOMBINANT BCG VACCINE ON THE CURRENTLY USED BCG

What are the implications for the current BCG vaccine if VPM1002 enters the market in the next 5 to 10 years? We should not forget that BCG is an effective vaccine for the prevention of TB disease for many individuals. Enthusiasm for new vaccines must not overshadow support for an existing, effective vaccine. Globally, there have been shortages in the BCG vaccine supply since 2013, and the situation appears to be worsening, with a shortage of 8 million doses in 2014 and 17 million doses in 2015 (17). The BCG shortage has highlighted the shocking fragility of the global manufacturing capacity for the only available vaccine against TB, the world’s leading cause of death due to a single infectious agent. It is not clear who should take responsibility for rebuilding manufacturing capacity and protecting our BCG supplies for the future. We are many decades away from seeing a recombinant BCG vaccine replace the current BCG vaccine for global use. Perhaps we should be asking ourselves if, while building capacity for the manufacture of recombinant BCGs, we also have a moral responsibility to support and rebuild capacity for the manufacture of the current BCG vaccine.

## SUMMARY

In the next 5 to 10 years, the recombinant BCG vaccine BCG Δ*ureC*::*hly* (VPM1002) may be the first TB vaccine to enter the market since BCG was first used in 1921. Like Sleeping Beauty, we have slumbered for 100 years with the current BCG vaccine, showing a reluctance to invest in clinical development and neglecting to secure existing biomanufacturing capacity. BCG Δ*ureC*::*hly* Δ*nuoG* is our wake-up call, showing us that we can and should make better vaccines with continued effort. TB vaccine development requires scientists to be awake and alert so that the introduction of the first recombinant BCG vaccine accelerates and does not stop the development of 2nd- and 3rd-generation vaccine candidates. Only through protection of our current BCG vaccine stocks and continued investment in preclinical and clinical development can we hope to reduce the global burden of TB disease.
